# Accuracy of point-of-care ultrasound by pediatric emergency sonologists for the diagnosis of skull fractures

**DOI:** 10.1186/2036-7902-4-S1-A4

**Published:** 2012-12-18

**Authors:** Joni Rabiner, LM Friedman, H Khine, JR Avner, JW Tsung

**Affiliations:** 1 Department of Pediatrics, Division of Pediatric Emergency Medicine, Children’s Hospital at Montefiore / Albert Einstein College of Medicine, Bronx, NY, USA; 2 Department of Emergency Medicine, Division of Pediatric Emergency Medicine, New York, NY, USA

## Background

Head trauma is one of the most common childhood injuries, accounting for > 600,000 emergency visits annually. It is estimated that 16% of children with nontrivial head injuries may have skull fractures. There are a number of studies supporting the use of point-of-care ultrasound (PoCUS) in fracture diagnosis. However, there is limited data on the role of PoCUS for skull fracture diagnosis.

## Objective

Our objective was to determine the test performance characteristics for PoCUS performed by pediatric emergency medicine (PEM) physicians compared to CT diagnosis of skull fractures.

Patients and Methods: We conducted a prospective convenience cohort study of patients up to 21 years of age who presented to two urban, level II trauma pediatric emergency departments with head injuries and suspected skull fractures requiring CT scan evaluation. After a 1-hour focused US training session, PEM attendings and fellows performed US examinations to evaluate for skull fractures. CT scan interpretations by attending radiologists were the reference standard to determine test performance characteristics of skull US. PoCUS scans were reviewed for errors by an experienced sonologist.

## Results

PoCUS was performed on 72 subjects with suspected skull fractures by 17 PEM physicians. The mean age was 6.5 years (SD 6.2 years) and 67% of patients were male. History and physical exam findings included scalp hematoma in 63%, vomiting in 31%, loss of consciousness in 13%, Glasgow coma scale < 15 in 11%, and palpable fracture in 7%. The prevalence of fracture was 11.1% (n=8). PoCUS for skull fracture had a sensitivity of 88% (95% CI 53-98%), specificity of 97% (95% CI 89-99%), positive likelihood ratio of 28.0 (95% CI 7.0-112.3), and negative likelihood ratio of 0.13 (0.02-0.81). The only false negative scan was due to a skull fracture not directly under the scalp hematoma, but rather adjacent to it. US scans took a median of 65 seconds (IQR 35-139 seconds) to perform.

## Conclusion

PEM physicians with focused US training were able to diagnose skull fractures in children with high specificity.

